# Filling the Void: The Role of Adult Siblings Caring for a Brother or Sister With Severe Mental Illness

**DOI:** 10.1177/23333936231162230

**Published:** 2023-03-30

**Authors:** Gabriele Kitzmüller, Lena Wiklund Gustin, Anne Martha Kalhovde

**Affiliations:** 1 UiT, the Arctic University of Norway, Narvik, Norway; 2 Mälardalen University, Västerås, Sweden; 3 Jæren District Psychiatric Center, Bryne, Norway

**Keywords:** family, mental illness, narrative, roles, siblings, Norway, familie, narrativer, psykisk lidelse, roller, søsken, Norge

## Abstract

Little is known about the role of adult siblings’ caregiver role within the context of mental illness. Therefore, our purpose was to explore how siblings narrate their experiences of being the main caregivers of a brother or sister with severe mental illness and how they cooperate with their ill sibling and their family of origin. We used a narrative hermeneutic approach and performed a secondary analysis of two interviews of siblings derived from a study of peoples’ experiences of hearing voices. The findings illuminate the participants’ multifaceted roles and how differently siblings might deal with the multiple challenges of caring for an ill sibling. The mediating role between their ill sibling and their family of origin to reestablish the broken family bonds was a significant aspect. Nurses’ awareness of the important and multidimensional role of caregiving siblings can improve the provision of family support and promote involvement of siblings in the treatment of an ill family member.

## Abstrakt

Vi vet lite om hvordan voksne søsken ivaretar rollen som omsorgsgivere når et familiemedlem har en alvorlig psykisk lidelse. Hensikten med denne studien var derfor å undersøke søskens narrativer med tanke på hvordan de erfarer omsorgsrollen og hvordan de samarbeider med den som har en psykisk lidelse og med resten av familien. Vi foretok en hermeneutisk sekundæranalyse av narrativene i to søskenintervju fra en studie som utforsket erfaringer med det å høre stemmer. Funnene synliggjør deltakernes mangefasetterte roller og hvordan de møter utfordringene med å yte omsorg for en person med alvorlig psykisk lidelse. Et fremtredende funn var at de inntok rollen som brobygger i et forsøk på å gjenopprette brutte familiebånd. Det er betydningsfullt at sykepleiere er oppmerksomme på de sammensatte rollene søsken kan ha som omsorgsgivere. Sykepleiere kan fremme involvering av søsken og forbedre familiestøtte i behandlingsforløpet til de som har en alvorlig psykisk lidelse.

## Introduction

The families of people with serious mental illness (SMI) play a prominent role in caring for an ill family member. Parents are usually the main caregivers. When they are no longer capable or for different reasons able, siblings frequently take over the caregiver role for an ill brother or sister (Hatfield & Lefley, 2005; Leith et al., 2018). We know a great deal about sibling relationships in general, and families with SMI in particular, but less is known about how adult siblings perceive their role as main caregivers for a brother or sister with SMI.

Sibling relationships are often the longest lasting relationships within families (Milevsky, 2016; Whiteman et al., 2011) and sibling commitment and emotional support often remains stable across the lifespan (Rittenour et al., 2007). This also means that they become significant people in each other’s narratives, and thus contribute to the construction of their narrative identity (Ricoeur, 1984). Sibling relationships maintain a number of different roles and are portrayed as the place where people learn that conflicting emotions can exist within the relationship (Sanders, 2004).

The quality of the sibling relationship has an impact on cognitive, emotional and social development during the life span (Milevsky, 2016). Stress in families may weaken sibling bonds (Sanders, 2004) and siblings’ relationships are more at risk after negative life events, including mental illness (Voorpostel et al., 2012). The quality of the sibling relationship is threatened especially, if the ill sibling shows deviant behavior or violence (Al-Sawafi et al., 2021; Bowman, Alvarez-Jimenez, Wade, Howie et al., 2014; Sporer et al., 2019) or has ongoing substance use (Bowman, Alvarez-Jimenez, Wade, McGorry et al., 2014).

Siblings living in families with a brother or sister with SMI experience numerous challenges and emotional strain when dealing with ill siblings’ symptoms (Lukens et al., 2004). Overall, those siblings’ emotional (W. Y. Chen & Lukens, 2011), psychological well-being (Taylor et al., 2008) and quality of life (Bowman, Alvarez-Jimenez, Wade, McGorry et al., 2014) are at risk. In addition, well siblings often feel forgotten and neglected by their parents (Schmid et al., 2009).

Siblings of persons with SMI perceive high personal losses (Dulek et al., 2021; Leith & Stein, 2012; Stein & Wemmerus, 2001; Stein et al., 2020), even more so than their parents (Leith & Stein, 2012). On the other hand, siblings report more satisfaction with personal experiences and relationships than parents do (Al-Sawafi et al., 2021; Sin et al., 2016). Siblings experience depressive symptoms and high levels of grief and family stress (W. Y. Chen & Lukens, 2011). In addition a high number of siblings with a brother or sister with early psychosis have experienced suicidal ideation or attempts in their ill sibling leading to poorer quality of life (Bowman, Alvarez-Jimenez, Wade, Howie et al., 2014). Siblings regret loss of their normal life before the illness (Stein & Wemmerus, 2001) and loss of the former relationship with the ill sibling (Dodge & Smith, 2019; Kovacs et al., 2019; Sin et al., 2008; Stålberg et al., 2004).

In general, spousal and parental caregiving are perceived as more burdensome than caregiving for siblings or others (Pinquart & Sorensen, 2006; Viana et al., 2013). Overall, gender influences on how caregiving is performed and perceived (Hanlon, 2012; Sharma et al., 2016) with women experiencing more caregiver burden than men (Penning & Wu, 2016; Viana et al., 2013). Research on how siblings’ gender influences on caregiving in the context of mental illness is sparse. However, sisters seem to be more likely than brothers to engage in caregiving (Chevrier et al., 2022; Gerstel & Gallagher, 2001) and they show significantly poorer mental well-being compared to normative scores (Sin et al., 2016).

Living with a sibling with SMI may call forth negative emotions toward the ill sibling and influence negatively on well siblings’ self-perception (Barnable et al., 2006; Lukens et al., 2004; Sin et al., 2008) and self-care attitudes (Stein et al., 2020). Overall, caregiving siblings report poor communication with and support from health care services (Lukens et al., 2002). They express a need for information and psycho- education to maintain their own well-being and to provide ongoing support to their sibling (Sin et al., 2014, 2017).

Nevertheless, caring for a family member with mental illness is grounded in love, affection and commitment and arouses feelings of empathy and compassion and a willingness to provide ongoing support (Lashewicz et al., 2012; Shiraishi & Reilly, 2019). Siblings report deep bonds to their ill brother or sister and feel satisfaction and fulfillment in their caregiver role (Dodge & Smith, 2019; Schmid et al., 2009). In addition, the caring relationship may enhance the well siblings’ personal growth (Stein et al., 2020) and resilience (Sin et al., 2008).

A recent systematic review and qualitative meta-summary shows that conflicts in interpersonal relationships and family disruption are common in families living with SMI (Shiraishi & Reilly, 2019). We know little about how those families deal with conflicts and family disruption and even less about how the well siblings position themselves in those families, other than experiencing confusing role shifts from being a sibling to playing the role of a parent (Kovacs et al., 2019). We assume that the long, close and simultaneously challenging relationship contributes to the caregiving siblings’ understanding of themselves along with their significance and responsibility for their ill sibling, in other words aspects of their narrative identity (Ricoeur, 1984).

### Purpose and Research Question

To extend the existing knowledge on adult siblings’ roles our purpose was to explore how siblings narrate their experiences of being the main caregivers of a brother or sister with SMI and how they cooperate with their ill sibling and their family of origin. Thus, our research questions were: What do adult siblings’ narratives reveal about their role as main caregivers? How do they position themselves within their family of origin and engage in the challenges of having a sibling with SMI?

### Norwegian Context

The average Norwegian family has a nuclear structure (Solheim, 2012). The obligations of care are usually maintained within those nuclear families engaging both men and women (Ellingsæter, 2014). Although, women take part in work life almost as much as men, caring obligation is the reason why women take on part time jobs more often. Nevertheless, local authorities have the legal responsibility for care in Norway (Mestheneos & Triantafillou, 2005).

In recent years the number of single families and single parents’ families has increased (Innstrand et al., 2010). As Norway is a wide stretched country consisting of extensive and sparsely inhabited rural areas, adolescents and young adults often have to move on to urban areas for the purpose of education or access to the labor market (Bæck & Paulgaard, 2012). In addition, a high divorce rate in Norway leads to the establishment of new partnerships during the life course (Nilsen et al., 2018). These changes in traditional family patterns may add to changed patterns in caregiving. Approximately 800,000 persons in Norway act as informal caregivers of their ill family members at any time (Regjeringen, 2021). Totally 136,000 man-labor years are spent on informal caregiving in Norway annually (Hjemås et al., 2019).

Statistically there are no significant gender differences in caregiving in Norway as men’s share in providing care has increased rapidly during the last years (Regjeringen, 2021). Nevertheless, time spent on caregiving tasks differ between gender, with women providing most care for parents and men providing most care for other family members and persons in their network (Regjeringen, 2021). Studies on how gender influences on siblings’ caring patterns are missing in Norway as elsewhere.

Persons may warrant any family member or close friend to get access to information and the right to contribute to decision making regarding their treatment and care in the Norwegian health care systems, especially in case they are not competent to make decisions on their own (Lovdata, 1999). Guidelines for health care workers on how to involve significant others have been established recently in Norway (Helsedirektoratet, 2017).

In the past 20 years resources within mental health care have increasingly been channeled from specialized health care to community health care in Norway (Norwegian Ministry of Health and Care Services, n.d.; St.prp.nr. 63, 1998). This has led to a shift from long-term in-patient treatment to shorter admissions and out-patient treatment at local district psychiatric centers along with various community health care services. The Norwegian authorities have initiated several campaigns addressing more openness about mental health, illness and stigma. The latest official strategi addresses mental health for the entire population, including family carers (Regjeringen, 2017).

### Theoretical Concepts of Caring

In accordance with Pearlin et al. (1990, p. 583) we define family caregiving as the behavioral expression of one’s commitment to the welfare of another family member. Usually, the illness of a family member puts challenges on the life course of both the ill family member and other close family members (Chibucos et al., 2005). The transition to caregiving is seen as one of the most challenging phases of caregiving, often leading to emotional turmoil and decline of well-being in caregivers’ lives (Marks et al., 2002). Nevertheless, transition to caregiving may have positive outcomes for the caregiver, adding to personal growth, purpose in life, self-acceptance and pride (Shiraishi & Reilly, 2019; Stein et al., 2020).

Researchers have suggested to put on a dyadic lens when studying caregiving as it involves both the one who is in need of care and the caregiver, as well as the relationship between them (Ferraris et al., 2022; Revenson et al., 2016). Both parts experience illness together and their coping abilities are put on trial and may influence on the illness trajectory (Shaffer et al., 2020). The quality of their relationship and the ability to communicate effectively will influence on how illness is managed collaboratively (Sud et al., 2021). Additionally, personality traits will influence on the care giver’s skill to provide care and to deal with burden and on the care recipient’s ability to accept care as beneficial (Revenson et al., 2016).

Lynch (2007) states that care work involves emotional, mental, physical and cognitive work as well as moral commitment that is strong and compelling. According to Lynch the inner circle of caregiving is defined by intimate caring relationships within the family, so called love labor. It is defined by strong attachment, commitment, responsibility and trust. This inner circle is surrounded by outer circles of secondary and tertiary care work, performed by network, friends or by means of statutory obligations. Lynch states that love labor is not commodifiable as it is voluntary in nature. Especially in the context of illness it provides a source for developing strong bonds between family members that may bolster the ill family member’s coping resources.

## Methods

### Study Design

We performed a secondary analysis of existing data originating from a hermeneutic phenomenological study of peoples’ experiences of hearing voices (Kalhovde et al., 2013, 2014). A narrative hermeneutic approach inspired by Ricoeur’s (1976) philosophy was chosen to interpret the meaning of the participants’ lived experience of their roles as siblings when caring for a brother or sister with SMI (Wiklund et al., 2002).

In the primary study (Kalhovde et al., 2013, 2014), the data from family members were found to be insufficient regarding the aim: how immediate family members of ill persons perceived living with the phenomenon of hearing voices. However, the primary analysis did indicate rich descriptions of siblings’ roles as main caregivers, which is an underrepresented research area. A secondary analysis allows the researcher to pose new research questions to an existing data set (Thorne, 1998), and may invite new researchers uninvolved in the original study, to participate in the re-analysis of data (Heaton, 2008). In this way, important information from descriptively rich, yet underutilized qualitative data sets can be drawn (Hinds et al., 1997).

The third author Anne Martha Kalhovde, who had collected and analyzed the original data, invited the first author Gabriele Kitzmüller to join the present study. The motivation was that investigation by another researcher with a different perspective might provide new insight on data that received insufficient focus in the primary study (Ruggiano & Perry, 2019). Kitzmüller’s experience in research on family members of people with chronic illness led to new research questions regarding siblings’ roles in relation to a family member with SMI. The second author Lena Wiklund Gustin was invited based on her expertise in psychiatric care and phenomenological-hermeneutic research.

### Participants and Sampling Strategy

In the primary study, seven participants had been recruited by participating family members with SMI, local groups of the national association for relatives in mental health and by reports in newspapers. The two texts we chose for the secondary analysis were the only transcripts deriving from siblings. We recognized that the siblings’ texts differed from the other five interview texts of family caregivers (parents, spouse) and held potential for new knowledge about adult siblings’ family roles as main caregivers.

Alice was an elder, single, sister in her forties, while Jon was a younger brother with a family of his own in his thirties. Both were ethnic Norwegians, had vocational education and held full time jobs with a low to average income. Alice had work experience within health care. Alice’s ill sibling lived with her and Jon’s lived nearby. Both had functioned as the main caregivers for their ill sibling for at least 10 years. The siblings with SMI, Eric and Linn, were in their forties and had vocational education and some work experience. They had received a schizophrenia diagnosis and used neuroleptic medication for more than 10 years. Linn struggled with additional substance use. Both had had multiple hospital admissions, several of them involuntary, and received community mental health care services, such as supported housing, supported work and home visits. Both ill siblings received disability pension. Both families consisted of several siblings, which lived in different parts of Norway. Alice’s and Linn’s parents were deceased. Both families had issues that created conflicts, alienation and disrupted communication.

### Data Collection

The interviews had been digitally recorded and transcribed verbatim by the third author. One interview had taken place at her workplace and the other in the participant’s home. No other persons had been present during the interviews. The interviews lasted 80 and 120 min. The third author encouraged the participants to convey their experiences of relating to a family member with SMI and posed follow-up questions to clarify details to ensure mutual understanding. She aimed to engage the participants in a dialogical hermeneutic learning process, in which she could get to know something new from the participants and not merely confirm her own preunderstanding.

### Ethical Considerations

The Regional Committee for Medical and Health Research Ethics in Northern Norway (P REK NORD 2010/3064) and the Norwegian Social Science Data Services (NSD 15313) approved the original study. All participants had given written informed consent. All procedures complied with the Helsinki Declaration (World Medical Association, 2000). The third author anonymized the transcripts prior to the secondary analysis. Additional steps were taken to avoid identification of participants. Therefore, certain characteristics have been omitted and others changed. All names are pseudonyms.

### Data Analysis

The narrative hermeneutic analysis was inspired by Ricoeur’s (1976) theory of interpretation and was developed by Wiklund et al. (2002). This method of analysis has been evaluated and compared with other Ricoeur inspired analyses earlier on (Singsuriya, 2015). After extracting data from the two interviews that had relevance for the re-analysis, we performed the analysis in three steps. These steps were an initial (naïve) interpretation, an analysis of narrative structures and a comprehensive interpretation.

In the naïve interpretation, we read the interview texts several times to gain an initial understanding based on our different assumptions of what the narratives conveyed about siblings’ roles. We attempted to understand these written interpretations from our different horizons of understanding. During the structural analysis, we sought for narrative structures in the texts, first by focusing on the way the narratives were structured around a plot, thereafter by searching for a deeper structure of the texts in the form of a metaphor that was idiomatic for both texts (Wiklund et al., 2002). In the last step of the analysis, findings from the previous steps were related to each other and to previous research to arrive at a new and broader understanding of siblings’ roles when caring for a brother or sister with SMI. This hermeneutic process between the parts and the whole enables alternative and more nuanced understandings of the text (Wiklund et al., 2002).

## Results

### The Narratives

To give voice to the participants and facilitate the reader’s understanding of the whole, we will present each sibling’s narrative before presenting the results of the analysis. As people tend to move back and forth between different episodes in an interview, we have organized the data extracted from the original interviews as coherent narratives representing the whole.

#### Jon’s Narrative

Jon was in his mid-twenties when he had to take care of his brother Eric with SMI. Although the brothers had parents and other siblings living elsewhere in the country, Jon was the only one who kept in touch with his ill brother living nearby. Previously, Eric had rejected his parents, and the other siblings distanced themselves from him. An acute psychotic episode required Jon to take action and to engage much more in his brother’s life. He brought Eric to his own apartment and spent several days with his seriously ill brother who suffered from hallucinations, delusion and paranoia. Jon realized that he had to send his brother to a psychiatric ward against his will and described this agonizing experience:

It was me who turned him in for the first time. It got very intense. I had to push him to his limits to confront him with his situation. At the same time, I had to comfort him. At the end, I managed to change his point of view and still hold on to his confidence in me. It took me years to get over that incident. For a long time, I could feel it in my body. There was no debriefing, nobody talked to you about that. It was an acute situation and I had to keep my head cool and my heart warm to get through it. When there’s no need to keep your head cool any longer, you realize what’s going on. I was lucky to have competent friends to talk to. Especially one friend who studied psychology supported me. He helped me to see that my brother had a life of his own and I had mine. I had to protect my own freedom and I shouldn’t be afraid of what might happen tomorrow, if he should kill himself.

Later during the interview, it turned out that Jon spent much time with his brother trying to support his therapy and striving to understand the impact of his illness. Although Jon had joined his brother during his therapy sessions for years, he underlined that he still understood very little of his illness symptoms.

During the past four or five years, we’ve had contact once a week or twice a month. Once a month we see a therapist together and for the last four or five years we’ve been to group therapy together. Although we have a good relationship, I know very little. Towards me he doesn’t put a lid on anything he’s thinking about, but what’s going on inside him, I don’t know. It’s difficult for me to distinguish what is him and what are his voices. I don’t know if the voices make him withdraw, silence him or make him say something that doesn’t fit in. It’s still like I understand very little of it. They [*voices*] are very dominating and they torture him. I’m standing outside and I cannot hear them but I can see his strong reactions. He withdraws and there are periods of substance abuse.

The interview text revealed that Jon had an important mediating role within his family. He had to mend the broken bonds between Eric and his parents and other siblings as well as Eric’s ex-wife and teenage son. Eric’s encounters with his son were dependent on Jon’s presence.

As a brother, I know far too many private and intimate things about Eric. Things you shouldn’t know as a brother. I have to keep a balanced distance, and I tell very little to the rest of our family. This is in agreement with my brother who doesn’t want them to know anything about his situation. On the other hand, I have a mother whose greatest wish is to have contact with him. I’ve told her a bit more than I’m allowed to. I don’t mention suicidal attempts and such things. There is a geographical and relational distance and the information I give has to be adjusted. To hear about bad things isn’t good if you haven’t had any contact for many years with a person you’re missing and thinking of 24/7. As a mother, you should feel good and believe that things are getting better. She’s been grieving a lot. She’s given him many chances and has tried to support him. Obviously, she’s got a guilty conscience for a thousand things. But she also understands that it’s important for me to protect my relationship with him. I’ve told them about my role and my agreement with him that I don’t tell anything. Still, they get to know a bit more than that. I want all of them to feel all right.

In addition to his caregiver obligations, Jon has to take care of his own family. Fortunately, Jon’s partner has a good relationship with his brother. “My fiancée is a fantastic person. We discuss a lot and we find solutions together. She has a close relationship with my brother.”

Jon clearly also held an important mediating role in Eric’s new family and the health and social care workers involved in Eric’s treatment and care.

I played an active role when Eric lost custody of his son. I contacted child welfare and said they had to put him in care. I stated quite openly to Eric: ‘You’re not capable of taking care of your son.’ I think it’s the best thing to maintain a stable relationship to say what you think, what you’re going to do and then do it. I’m the boy’s uncle, I’m the link between him and Eric. Their relationship is important to me, his son is. As long as he says and does things that aren’t good for his son, I have to talk seriously with my brother. It happens that he gets worse from these conversations, more psychotic, but the most important thing is that his son feels ok. He’s innocent, my brother isn’t.

Several times during the interview, Jon emphasized the importance of family bonds and his respect for his elder brother.

My brother often asks me if he’s a burden to me, but he’s part of my family, you know. I tell him: ‘We aren’t together because you need my help. You can cope, but we’re together because we’re family.’ I introduce him as my big brother and it’s important to show that nobody can take this privilege from him. There are special constellations and relationships within a family, some members are older and some are younger. On top of that, my brother is ill and needs me, and he considers himself a burden. He’s my inferior, so it’s important to keep the balance and let him feel that he still ranks above me [*as the older brother*]. Still, that doesn’t mean I do everything to please him. If I don’t agree with him. I speak up to him and make my demands clear.

Jon’s role as caregiver for his ill brother involved strong moral obligations and feelings of inadequacy: “I have a guilty conscience because I can’t be there for him enough. I’m not always available. You make an appointment and then you have to cancel and say, ‘Sorry I couldn’t make it.”

#### Alice’s Narrative

Alice had taken care of her younger sister Linn with SMI since they were young adults. The sisters had grown up with their parents and many other siblings. Alice had closely monitored Linn’s treatment and care through the years. She had constantly fought for her ill sister’s rights, to improve her physical and mental health, her treatment and her living arrangements. Several times, she had intervened when professional care had failed. Recently, Alice had rented an apartment with Linn, as the living arrangement provided by the municipality had not worked for her sister. Alice felt the responsibility for Linn’s well-being on her shoulders and sometimes she longed for professional support. Lately, she had found that health care workers acknowledged her expertise as a close family member and had listened to her advice. Despite her struggles, Alice had maintained a positive view of life and emphasized her sister’s resources rather than her weak health:

It’s important for her improvement that she’s aware of her possibilities. My aim as her sister is to give her a good life, so we can avoid those acute relapses. Because those days when she was extremely ill it was a tremendous struggle. Now she hasn’t had any acute admission for years. She only has planned admissions, twice a year. There’s been a tremendous change from being in an institution for months and living in a sheltered accommodation for years.

Alice considered her sister’s symptoms as natural and felt that they might also occur in healthy people. Nevertheless, she recognized her sister’s struggles and actively tried to interpret her symptoms in order to understand them. Alice tried to alleviate her sister’s suffering by entering into her imaginary world and by driving out her demons.

Linn experiences them [*the voices*] as real and I must take her seriously. She hears a voice of a child crying. It hasn’t happened recently but previously it went on for years. I wondered if it had something to do with her own childhood but she cannot remember. I tell her: ‘Maybe you can talk to this child inside yourself.’ We have an advantage because we can talk about our memories. When we’re working through that, keeping a positive attitude, the sad, miserable and scary memories decrease. I think my way of dealing with things has helped her. We’ve worked through the heavy stuff together. I believe in sorting out things, facing the fear. She used to say to me: ‘Sis, you have to throw out this man, because there’s a man here’ and I told her: ‘Yes, I’ll send him up to the light.’ It’s my way of dealing with it.

Alice had been the first person to learn that Linn had been abused as a child by a close relative. For Alice it meant a lot that her sister trusted her. It had taken time for Linn to realize that her sister supported her as best she could during childhood.

There had been some dark spots in my childhood too. As a child, I’d observed things and realized that something wrong was happening. I remember Linn wanted to stay in my bed. I can recall asking her: ‘Can’t you go to your own bedroom?’ But she didn’t want to. She told me she’d been angry with me because she wondered why I didn’t help her. I told her: ‘We have to put that behind us. I was a child myself. You cannot accuse me of not helping you. I didn’t even understand what was going on.’ Every time she left my room, she’d been in danger. My parents hadn’t been present all the time. We had to talk through those things. That incest thing was a difficult subject. It was very bad for her self-esteem and brought out a lot of negative feelings. We had to clear out those things and put mutual trust instead. She knows she can trust me. After she told me about it [*abuse*] I kept it to myself for five years until she let me tell the other brothers and sisters.

Alice told about her own mediating role between her ill sister and the other siblings and how she confronted them with the fact that Linn had been abused by a family member.

After our parents died, we invited all of them to a family meeting. I confronted them with the truth and I confronted our relative with what he’d done. After that, we split up. Half of them thought I’d lied. We dropped the case for a while but in the end, they realized that I myself had felt that something wrong was happening. Some of them felt ashamed, they felt threatened when Linn had to see psychologists. Because then they had to touch on it themselves. I believe in bringing the whole truth to light. It’s not to condemn anybody, but she [*Linn*] couldn’t go on like that. We had to spend years building up her confidence to feel safe in her childhood home where the abuse had taken place. To begin with, she got worse when she’d visited our parents’ home. The voices took control over her. She was weak then and her self-perception was low. We had to build up a safe platform and it worked.

Another important role Alice took on was to teach the other siblings about Linn’s illness and to advise them on how to treat her.

I explained to one of our brothers [*when Linn was staying with him*]: ‘She needs structure, she mustn’t sleep all day. You have to be patient with her and give her time.’ One of our sisters panicked and thought she had to hide all her knives [*when Linn visited her*]. We had to talk about it and finally, we all laughed. I told her: ‘If your sister wants to kill herself, you’d better hide everything she might use. Then there’ll be nothing left in your apartment.’

Alice gave a vivid description of her strong family bonds toward her sister and the reason why she was willing to maintain her role as caregiver for Linn.

Some people tell me: ‘You have to realize that she’s a burden for you.’ But I don’t agree. She’s a resource. I believe in the power of love; we are bound together by these bonds. It’s our togetherness, she’s a resource for me too, she’s my sister, we’re family. I’m a person who’s focused on family values. I think it’s important to build your life on this platform. I don’t want to focus on her illness and on the things that turn out wrong. I’m focusing on making it work.

### Naïve Interpretation

Despite coming from large families, Alice and Jon are the only caregivers available to their ill sibling. The other family members are either unable or unwilling to take on the role as the main caregivers or they have been rejected by the ill person. Confronted with conflicting demands and dilemmas, Alice and Jon are pulled in different directions when trying to bring their family together. While maintaining their role as siblings, they are forced to take on parent-like roles that demand considerable time, energy and responsibility. Both siblings act as mediators, using different strategies to prevent earlier conflicts from flaring up again. They perform their mediating role by means of confrontation, open dialog or by avoiding sharing potentially harmful information. Both Jon and Alice are engaged in the treatment of their ill sibling and they try to help them to gain control over their recovery process. The role of the main caregiver for a person with SMI demonstrates how strong family commitment is associated both with love and emotional drain.

### Narrative Structure

In contrast to other researchers, such as Lindseth and Norberg (2004) who focus on thematic analysis in their structural analysis, Wiklund et al. (2002) focus on narrative structures, or what Ricoeur (1984) describes in terms of emplotment. According to Polkinghorne, the plot of a narrative clarifies the point of the story, the lesson to be learned and the meaning embedded in the story (Wiklund et al., 2002, p. 119). In line with Ricoeur’s description of the structural analysis as an explanatory step, we argue that different narrative structures of how the plot is presented reveal different things about the storyteller. Thus, focusing on how the text is structured around the plot is a means to explain the text as text, and thus also an act of distanciation (Ricoeur, 1973, 1991).

The concept narrative identity contributes to an understanding of participants’ situation as the concept acknowledges that our identity is constructed in the interplay with others. This interplay is not only on a behavioral level. According to Stern (1990) our sense of self is partly based on how we narrate, and thus ascribe meaning to our personal experiences during our interplay with others. By acknowledging narrative identity and by analyzing how participants structure their narratives by means of emplotment we also strive to show consistency between methodological and theoretical aspects.

We examined the narrative structure of Jon’s and Alice’s stories. In this step of the analysis, we looked at how the narrative is structured around the plot, and how the narrator positions him/herself in relation to the plot. As described by Gergen and Gergen (1986), the way we form and structure our personal narratives is closely associated with how we make sense of events over time. Hence, this interpretive step is adapted to add nuances to the spoken narrative, by focusing on both what is narrated and how it is narrated. White (1973) describes four types of narrative structures in our Western culture: tragedy, satire, comedy, and romance. When a narrative follows the structure of a tragedy, the narrator portrays himself as a victim, whilst the narrative structure of a romance places the narrator (the main character) in the role of a hero who copes with challenges. In examining Jon’s and Alice’s stories, we found that both narratives were structured in a romantic mode (White, 1973; Wiklund et al., 2002).

The narrative structure reveals Alice and Jon as central agents within their narratives who cannot and will not turn away from their seriously ill sibling. Both have to deal with multiple and complex caregiving challenges that unfold over a long-time span. Choosing not to abandon their sibling requires their involvement in difficult decisions. Although they find caregiving demanding, they perceive their roles as meaningful and developing. However, the narratives reveal that they have different strategies to face the challenges they meet. A strong thread of commitment is visible in both narratives. The narrators take on demanding family obligations without question when performing their roles as family caregivers. Alice shows total commitment by inviting her sister to move in with her, while Jon keeps the distance that allows him to mediate in relation to his family of origin and his brother’s family of procreation. In both narratives, these siblings’ resilience is clearly visible. In spite of past challenging events and the protracted burden of supporting their sibling, Alice and Jon manage to combine these caring obligations with their own life projects.

### Root Metaphor

According to Ricoeur, metaphors can explain the deeper meaning of a text by shifting the meaning of words from a literal to a non-literal one (Ricoeur, 1974, 1984, 1991). Metaphors can thus be used to highlight different themes that arise during interpretation, in other words “parts” of an interpretation. A root metaphor aims at illuminating the underlying meaning of a text or a discourse. Alvesson et al. (2017) further describe that the articulation of a root metaphor, as well as narrative structures, play an important role in existential hermeneutics. Hence, in this study the root metaphor “filling the void” is used to illuminate the deeper, existential meaning found in participants narratives. A void can be interpreted as an unoccupied or deserted space that leaves an emptiness, a gap, a hollowness or a lack of something (Void, n.d.). Alice and Jon have the main roles in their family of origin to stand up for their ill family member. Their parents are dead or not in a position to or willing to act as caregivers. Instead of close family bonds, there is distance and alienation between family members. By stepping up for their ill sibling, John and Alice are trying to fill the void after the breakdown of relationships and communication in their families of origin.

Two other metaphors lead up to the root metaphor: “acting as glue” and thereby “mending the broken bond.” Due to the illness and other traumatic events the bond between family members is broken and has left a void in those two families. By acting as glue, these siblings link up the broken family bonds. In order to do so, they have to take on a mediating role in their families. Jon’s role is to keep their mother informed about Eric’s health condition and to act as an important link between Eric and his son. Alice’s role is to arrange family meetings to gain openness within the family and to give advice to other siblings how they should treat Linn. Acting as glue is challenging and filled with dilemmas for Jon and Alice. However, there is a power that strengthens them when trying to fill the void left in their families: the devotion, love and commitment they feel for their ill sibling.

## Comprehensive Understanding and Discussion

The first research question we posed was what siblings’ narratives reveal about their role as main caregivers. The naïve understanding, the narrative structure and the metaphors indicate the important caregiver roles these siblings take on when their parents are no longer able or willing to act as caregivers. The siblings carry out their caregiver roles from a different position than their parents, opening up for new opportunities, but also challenges. The caregiver role has become a major part of Alice’s and Jon’s life, demanding much time and effort, and often evoking a guilty conscience when they cannot spend enough time with their ill sibling.

The narrative structure reveals Jon and Alice as the central agents in their stories. Although the responsibility for the ill sibling is sometimes demanding, they both engage in treatment and care and take action to avoid exacerbation. Alice takes an active part in her sister’s therapy and helps her to deal with the traumatic childhood events to regain control over her symptoms and to develop her self-confidence. Previous researchers state that siblings should be included in therapeutic processes (Milevsky, 2020), as siblings’ supportive role during adulthood increases in importance (Volkom, 2006). While Alice describes her sister’s symptoms in a familiar way, Jon states that he still does not comprehend his brother’s illness although he can read Eric’s body language if symptoms are bothering him. The call for education and support for siblings of persons with SMI is well documented in the literature (Barnable et al., 2006; Bowman, Alvarez-Jimenez, Wade, McGorry et al., 2014; Leith & Stein, 2012; Sin et al., 2017).

The naïve interpretation reveals that Jon and Alice have adopted the role of the main caregiver for their ill sibling, a role that influences their life in many ways. They did so out of commitment, love and trust as described by Lynch (2007) as love labor. Nevertheless, caring for their mentally ill family member took its toll as previously documented (Lukens et al., 2004; Schmid et al., 2009; Shiraishi & Reilly, 2019). To maintain a relaxed relationship with their ill siblings, Jon and Alice had to reconcile themselves with their siblings’ suicidal thoughts and attempts. They had learned to perceive their siblings as responsible for their own lives and they avoided blaming themselves for their siblings’ actions as long as they did their best to provide support. Acceptance of mental illness as nobody’s fault has been found to be one of the most helpful coping strategies in siblings of persons with SMI (Friedrich et al., 2008).

Jon’s attitude of maintaining a certain distance to his brother in order to cope with his caregiving obligations is known as an important strategy from previous studies (Dodge & Smith, 2019; Friedrich et al., 2008). Distancing can be seen as a means to recharge one’s batteries and live one’s own life (Dodge & Smith, 2019). Nevertheless, distancing is sometimes perceived as emotionally draining (Karp & Tanarugsachock, 2000) and may evoke self-criticism and guilt (Kovacs et al., 2019).

The analysis demonstrates some of the emotional impacts of SMI on well siblings and other family caregivers known from previous studies (Leith & Stein, 2012; Lively et al., 2004; Shiraishi & Reilly, 2019). Jon reported the emotional roller coaster when he adopted the role of caregiver and the difficult decisions he made when referring his brother to a psychiatric ward against his will. He still feels guilty about being unable to live up to his own expectations regarding how much time he can spend on caregiving. Siblings’ feelings of guilt and self-criticism have been mentioned before (Barak & Solomon, 2005; Kovacs et al., 2019; Leith et al., 2018). Jon also has to set limits for his brother’s behavior as he must prioritize the well-being of his brother’s child and balance his efforts in order to maintain his role as caregiver. Lashewicz et al. (2012), who conducted a family case study with adult siblings as caregivers for a person with SMI and substance abuse, used the concept of ambivalence to describe the balancing act between loving devotion and the setting of limits to protect oneself from harm. Karp and Tanarugsachock (2000) describe similar findings in their study of emotion management in family caregiving in mental illness. Jon’s narrative reveals that he experiences ambivalence in two ways. He has to set limits to preserve his energy to continue caregiving. In addition, Jon has to take care of his own family and takes on the responsibility to protect his brother’s vulnerable child. Therefore, he has to balance the amount of time and energy spent on his brother, his own family and the family of his brother.

Studies exploring male caregiving show that men more than woman balance their caregiving activities toward their family of origin with caregiving activities within their own nuclear family (Herlofson & Ugreninov, 2014). Alice does not have other family commitments than caring for her ill sister. Not being married and having no obligations of one’s own seems to be an important factor for taking on caregiving duties (Revenson et al., 2016). In addition, single and childless siblings are known to rely more on each other and to have greater contact during their lifespan (Campbell et al., 1999).

Sibling relationships depend on the strength of emotional bonds between siblings (Connidis & Campbell, 1995). That makes it easier to understand the reasons why Alice and Jon and none of the other siblings in their respective families became the main caregivers. The interpretive metaphors reveal powerful sibling bonds and a strong sense of responsibility for the ill sibling that resembles a parental role. Milevsky and Heerwagen’s (2013) study shows that older siblings often feel responsible for their younger siblings and that parents expect them to act as role models, making it difficult for the older sibling to differentiate between the sibling role and the parental role. Siblings’ readiness to take on caregiver roles also depends on their parents’ practical and emotional support (Dulek et al., 2021). Smith et al. (2007), who explored siblings’ future expectations of caregiving for a brother or sister with SMI found that the quality of the relationship during childhood and adolescence, the seriousness of the illness and the geographical proximity of their homes influenced siblings’ expectations of future caregiving tasks. The quality of the sibling relationship is clearly seen in the naïve interpretation of Alice’s narrative. Although there are other brothers and sisters in her family of origin, Alice emerged as the caring and trustworthy elder sister during childhood and later in life. Seen through a dyadic lens (Ferraris et al., 2022; Revenson et al., 2016), the dramatic experiences during these siblings’ childhood may have strengthened their bond and enabled them to engage in a giving relationship for both. Alice’s narrative show that they were able to communicate openly within their dyad. In addition, Alice’s experience as a health care worker may have prepared her to engage in the dialog about her sister’s voices to support her coping resources and recovery process. It is supposed that the strong bonds and the love labor within the dyad have supported Lin’s coping resources.

Jon’s transition to caregiving led to emotional turmoil as described by Marks et al. (2002). He felt obliged to enforce his brother’s hospitalization, possibly out of a sense of responsibility for his brother’s future well-being. This is in accordance with the findings of Kovacs et al. (2019) showing that enforcement of the ill sibling’s admission to hospital can be the starting point of a long journey as main caregiver.

Further, the analysis reveals a positive appraisal of the caregiving situation despite the adversity. Alice’s deep involvement in her sister’s illness trajectory has contributed to her own personal development and growth. Caregiving as a possible source of existential growth and boundary stretching has previously been reported in family members of persons with SMI. F. P. Chen and Greenberg (2004) found that 50% of participating caregivers stated that caregiving had clarified their priorities in life and resulted in a greater sense of inner strength. According to the systematic review of Shiraishi and Reilly (2019) the positive impacts of caregiving such as affection, compassion, solidarity and personal growth, hold a central position within all the negative effects in families with severe mental illness.

According to Bandura’s social learning theory, family members are models for social learning and those who are kind and caring, competent or powerful are likely to act as role models (Bandura, 1977). The interpretive metaphors reveal that Alice and Jon both possess those qualities, and it is thus no coincidence that they have become the main family caregivers. Probably they have been good role models who could be trusted. Several studies emphasize siblings’ emotional closeness (Gillies & Lucey, 2006; Milevsky, 2020). In Milevsky’s (2020) study the similar or common life experiences and the amount of time siblings spent together are seen as drivers for this closeness. The findings in our study reveal that both siblings displayed emotional closeness to their ill sibling although they acted differently as caregivers. Alice moved in with her sister and showed a close and nurturing caregiver style while Jon adopted a more confrontational style and kept more distance. This difference may be understood in line with gender differences where men are known to use coping strategies such as detachment and distancing more often than women (Sharma et al., 2016). The differences we found may also comply with socially constructed gender roles in caregiving with women focusing more on the wellbeing of others than on their own needs (Bubeck, 1995, as cited in Hanlon, 2012, p. 39). Nevertheless, both siblings’ narratives reveal personality traits that have been reported as beneficial for caregiving, such as openness, a caring attitude, the ability to seek support and to plan ahead (McCrae & Costa, 2003, as cited in Revenson et al., 2016, p. 81).

Our second research question was how siblings of persons with SMI position themselves within their family of origin and engage in the challenges of having a sibling with SMI. Earlier research has pointed to the high level of conflicts and disruption in families with SMI (Shiraishi & Reilly, 2019). Knowledge about caregiving siblings’ role in those conflicts have been poorly described. The interpretative metaphors in our narrative analysis illuminate the void that the unfortunate and traumatic events left within the actual families, both before and after the onset of the illness. There was distance between family members that made it impossible to maintain communication between the ill family member and the others. In both families, one of the well siblings had taken responsibility to fill this void. Alice’s and Jon’s position in their families of origin may be seen as glue that enabled them to fill the void and to mend the broken bonds between family members.

Both participants mediate between the ill family member and the others to maintain some level of contact within the family. Although siblings’ mediating role within their family of origin has been briefly mentioned before (Schmid et al., 2009), it has not been described. The naïve interpretation reveals Alice’s approach as confrontational, whilst Jon’s approach is the one of a cautious mediator. He has to consider his concerned mother, and thus avoids sharing information that would upset her. Although he has promised his brother not to share any information about him with other family members, he cannot keep his promise entirely. Otherwise, he would not be able to act as glue, holding his family together. Siblings’ contradictory role of navigating between vagueness and confrontation in families with mental health problems has previously been described by Kovacs et al. (2019).

The naïve interpretation illustrates Jon’s perception of the hierarchical family structure of sibling relationships. He emphasizes the importance of respecting his elder brother’s authority. Although their roles have switched due to his brother’s ongoing need for support, it is difficult for Jon to deviate from this hierarchical structure. His normative expectation of birth-order roles agrees with the findings in the studies by Davies (2019) and Gillies and Lucey (2006), where support was described as passing from older to younger siblings due to the responsibility the older ones feel for the younger ones.

The narrative structure reveals Alice and Jon as resilient agents capable of engaging in the well-being of their ill siblings. Resilience is seen as people’s ability to use their resources to adapt to and overcome adversity and to avoid negative outcomes (Windle, 2011). In line with the description of resilience by Aburn et al. (2016), these two siblings show a strong ability to bounce back from the adversities of their negative family structures and to deal with disturbing experiences when acting as caregivers. Two literature reviews conclude that resilience depends on both the person’s individual resources and existing resources in the environment (Aburn et al., 2016; Windle, 2011). Similarly, Jon and Alice also have resources in their environment providing them with support. Jon’s shares his concerns openly with his partner who has a good relationship with Jon’s brother. Alice is a resource person in a peer group for family members of mentally ill persons, where she can share her experiences with others. The importance of peer groups as a resource for family caregivers has been documented earlier (Markoulakis et al., 2022; Sporer et al., 2019).

The analysis reveals Alice’s and Jon’s positive attitudes despite their challenging roles as caregivers. Both narratives reveal personality traits such as caring attitudes, openness, ability to seek support and an optimistic life view. These traits have been associated with better mental health in the caregiving situation (Koerner et al., 2009). Positive appraisal of a demanding situation has been found to promote resilience (Fortune et al., 2005). Alice and Jon are using open and sometimes confrontational communication strategies, which according to Jonker and Greeff (2009) are another crucial resilience factor in families dealing with mental illness. Contrarily, vague and indirect communication in those families may lead to unbearable inner conflicts and superficiality (Kovacs et al., 2019). An important precondition for family resilience mentioned by Jonker and Greeff (2009) is the loving and caring devotion for the ill family member, as clearly seen in the analysis, illustrated by Alice’s quote: “I believe in the power of love, we are bound together by these bonds.” Our findings support the conceptual framework deriving from a systematic review of caregiving in families with SMI, showing that caring attitudes reinforced by affection and compassion are essential when dealing with the traumatic experiences and negative impacts of caregiving (Shiraishi & Reilly, 2019). In addition, our findings provide nuances to the understanding of how it might be to care for a sibling with SMI, and thus allows nurses to come closer to participants’ experiences through the narrative approach.

### Study Limitations

According to Ricoeur (1976), the interpretation of a text should be the most plausible one, supported by the hermeneutical movement between the understanding of the parts and the whole of the text. We would argue that our different experiences and knowledge bases have given us a reflexive attitude and supported our endeavor to arrive at a valid interpretation. The third author used her field expertise and her knowledge of the interview context when analyzing the texts. The first author posed different questions to the text due to her expertise on family nursing. The second author who had developed the specific Ricoeur inspired analysis (Wiklund et al., 2002) searched for the narrative structure and contributed to the development of metaphors. Narratives are an important source of knowledge and reading narratives may enhance the reader’s appropriation of meaning. When translating the narratives, we have attempted to keep close to the perceived meaning of participants’ statements. A language expert was consulted during the translation process. We consider the close collaboration between the first and the third author in performing the analysis and its validation by the second author as a strength.

A challenge with secondary analysis is that the data are generated in relation to a different aim. Hence, it is important to reflect on what impact that might have had on the understanding of the narratives. As the original study was close to the current aim, and as the narratives about being the sibling of an adult with SMI are rich and vivid, we considered the data to be relevant to the aim of the present study. In a secondary analysis, the context of the interviews is often lost. In our study this was not the case as the third author conducted the interviews and shared this information with the team. Presenting participants’ statements from different parts of interviews as a coherent narrative may present a challenge as it may possibly gloss over participants’ experiences of caregiving as incoherent. Nevertheless, our participants presented their experiences quite structured with a clear emphasize on what they experienced as challenging within their role as main caregivers.

The small number of participants may be seen as a disadvantage. Nevertheless, both participants provided rich narratives, allowing for an in-depth understanding of their roles as siblings. Furthermore, the inclusion of each narrative in the manuscript was not only a means to give voice to Alice and Jon as two experts by experience. It will also enable the reader to validate the accurateness of the data for secondary analysis in a more profound way than with a traditional presentation with short quotes.

### Implications and Recommendations

Our findings demonstrate the importance of listening to siblings’ narratives that reveal their important role as caregivers, especially in cases where siblings are the main and only family caregivers. Knowing how siblings perform their caregiving role will make it easier for nurses to appreciate siblings’ contribution and to include them in planning treatment and care. Attentive listening will inform nurses about the support siblings need to strengthen their resilience and to avoid caregiver burden and stress. Assisting siblings of persons with SMI who act as long-term caregivers might be crucial for their health and well-being.

The findings suggest that some caregiving siblings still know very little about the illness in question and there is a need to adjust psychoeducation to their personal needs. In addition, psychoeducation programs and other targeted interventions will support siblings’ resilience (Zauszniewski et al., 2010). Better knowledge of siblings’ roles will make it easier for nurses to involve siblings in the treatment team. In addition, it will enhance the sharing of roles and responsibilities of different stakeholders, the lack of which has been documented (Pope et al., 2018). Future research should focus on developing and testing nursing interventions to support siblings’ resilience.

## Conclusion

In the aging population of Western countries, siblings must often care for a mentally ill brother or sister for many years. For nurses, it is important to listen to siblings’ narratives of caregiving to plan for their individualized support and psychoeducation. Caregiving siblings’ roles as “family mediators” need to be acknowledged. It is necessary to support families’ ability to communicate openly when facing adversity in caregiving. Often, adult siblings have a family of their own and must combine their caregiver role with their family roles as spouses and parents, in addition to working life. Society could be more explicit in appreciating the value of siblings’ contribution.
